# Area Deprivation and Social Vulnerability Are Associated with Pediatric Vision Screening Outcomes in the University of California, Irvine EyeMobile Program

**DOI:** 10.1038/s41433-026-04394-4

**Published:** 2026-04-01

**Authors:** Jainam Shah, John D. Hong, Kimia Rezaei, Jordan Tang, Michael Madsen, Cecilia Vallejos, Kourosh Shahraki, Kimberly R. Walker, Annabelle M. Storch, Joseph H. Bui, Jennifer Espinoza, Diana Torres, Donny W. Suh

**Affiliations:** 1 Gavin Herbert Eye Institute, University of California, Irvine, CA USA; 2 Albert Einstein College of Medicine, Bronx, NY USA; 3 University of California, Riverside School of Medicine, Riverside, CA USA

**Keywords:** Epidemiology, Education

## Abstract

**Background:**

The Area Deprivation Index (ADI) and Social Vulnerability Index (SVI) are nationally validated neighborhood-level measures of socioeconomic disadvantage but remain understudied in pediatric ophthalmology. We examined associations between ADI/SVI and school-based vision screening outcomes, refractive error (RE), best-corrected visual acuity (BCVA), and amblyopia suspect status.

**Methods:**

We conducted a retrospective study of children aged 3–10 years screened by the UCI EyeMobile program (Orange County, California, United States) from 2021–2024. Among 15,842 children screened, 3,350 underwent comprehensive examination. School addresses were geocoded to ADI/SVI quartiles (Q1–Q4; higher quartiles indicate greater disadvantage). Outcomes included referral, ‘fit-for-frames,’ RE, amblyopia suspect status, and poor BCVA (>0.2 logMAR). Group differences were assessed using chi-square and Cochran–Armitage trend tests. Multivariable regression evaluated associations with ADI/SVI after adjustment for age, sex, race/ethnicity, and RE type and severity. Race × index interactions were evaluated, and stratified analyses were performed when significant.

**Results:**

Children in Q4 neighborhoods had higher referral rates and were more likely to require new spectacles despite passing screening. Myopia was less common, whereas astigmatism was more common in Q4. After adjustment, both poor BCVA and amblyopia suspect status increased across quartiles (*p*-trend < 0.001). Compared with Q1, Q4 was associated with higher odds of poor BCVA (aOR [95% CI]: 1.51 [1.13–2.01] for ADI; 1.61 [1.18–2.20] for SVI) and amblyopia suspect status (1.78 [1.30–2.43]; 1.82 [1.33–2.48]). Significant interactions were observed, with stronger Q4 versus Q1 effects among Hispanic (aOR: 1.87) and Asian (aOR: 1.62) children, but not among Caucasian children.

**Conclusions:**

Children from the most disadvantaged neighborhoods had higher odds of amblyopia suspect status and poor BCVA. These findings support incorporating ADI/SVI in pediatric vision care to identify underserved children proactively, allocate resources equitably, and reduce the risk of permanent visual impairment.

## Introduction

Childhood visual impairment is a significant global public health concern. Uncorrected refractive error (RE), amblyopia, and strabismus are leading causes of pediatric vision loss, with estimates suggesting that nearly 20% of U.S. children have correctable REs and 2–4% are affected by amblyopia, the most common cause of preventable monocular vision loss [1–3]. Left untreated, these conditions impair visual development, academic performance, and long-term quality of life [4]. Despite the expansion of school-based vision screening programs, children from minority, low-income, and non-English-speaking families continue to face disproportionate barriers to timely diagnosis and treatment [5].

Growing evidence underscores the role of neighborhood context in shaping health outcomes beyond individual socioeconomic status (SES) [6, 7]. Traditional SES measures may not fully capture structural disadvantages such as poverty concentration, housing instability, or linguistic isolation [6]. Standardized neighborhood indices, including the Area Deprivation Index (ADI) and the Social Vulnerability Index (SVI), provide multidimensional frameworks for quantifying neighborhood disadvantage across several domains relevant to health and social vulnerability [8–11]. ADI measures socioeconomic disadvantage at the U.S. census block-group level (600–3000 residents), while SVI captures broader community vulnerability, incorporating SES, minority status, language, and housing/transportation at the census tract level (1200–8000 residents) [10, 11]. These different geographic scales offer complementary perspectives, as ADI identifies localized areas of poverty while SVI’s community-level scale alongside inclusion of minority status and language vulnerability may better capture barriers to healthcare access and continuity of care within racial and ethnic subgroups. Both indices are nationally validated and widely used in health equity and clinical outcomes research.

In ophthalmology, these indices have demonstrated disparities in glaucoma surgery and management [12, 13]. In pediatrics, higher ADI and SVI scores have been associated with reduced well-child visits, lower vaccination rates, and higher risk of obesity [14–16]. However, ADI and SVI have not been systematically evaluated in large-scale U.S. pediatric vision screening populations. To address this gap, we analyzed 15,842 children screened through the University of California, Irvine (UCI) EyeMobile for Children program, linking school-level geocoded data to ADI and SVI. By integrating both indices, we examined how neighborhood context intersects with race/ethnicity to influence screening outcomes, RE patterns, amblyopia suspect status, and corrected visual acuity (VA), including race–index interactions. We hypothesized that higher neighborhood disadvantage, measured by either ADI or SVI, would be associated with poorer vision outcomes and that SVI would demonstrate differential associations within racial and ethnic subgroups due to its inclusion of minority status and language vulnerability domains. To our knowledge, this is the first large-scale U.S. pediatric study to apply both ADI and SVI to vision screening outcomes, with direct implications for mobile eye programs and school-based initiatives serving underserved children.

## Methods

### Study design and setting

This retrospective cross-sectional study included children aged 3–10 years who participated in the UCI EyeMobile for Children program between January 2021 and December 2024. The program delivers in-school vision screenings and no-cost comprehensive eye examinations to underserved communities across Orange County, California, including preschools, Head Start centers, and elementary schools.

**Fig. 1:**
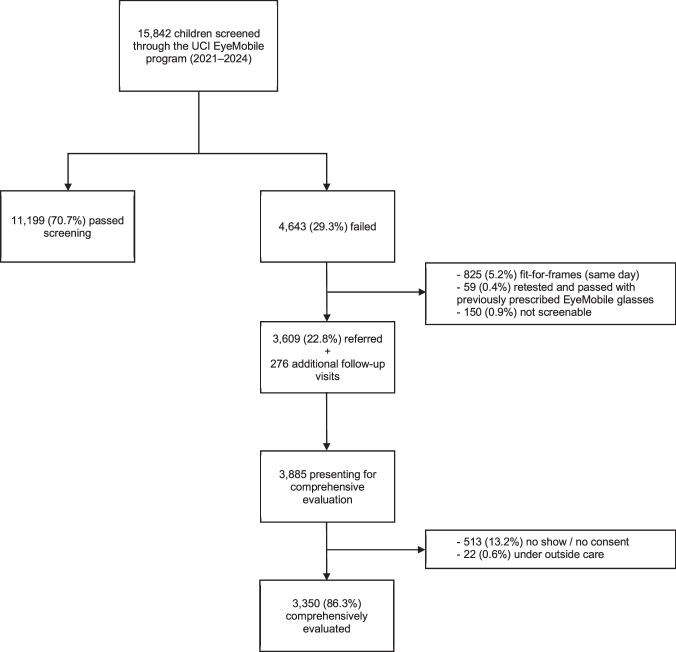
Flow diagram of screening, referral, and analytic cohort selection in the UCI EyeMobile for Children program (2021–2024).

Children were excluded if they were absent, already under outside optometric care, or lacked parental consent for follow-up examination. In total, 15,842 children were screened, of whom 3350 underwent full eye examinations (Fig. 1). The UCI Institutional Review Board (IRB) Committee provided an exemption from IRB approval as the study and retrospective analysis were not categorized as human subject research (IRB #1152). The study was conducted in accordance with the Declaration of Helsinki and the Health Insurance Portability and Accountability Act.

### Refractive error definitions

RE was classified using the spherical equivalent (SE), calculated as the spherical component (SPH) plus one-half of the cylindrical error (CYL). Measurements were obtained with the Retinomax K-Plus 3 Autorefractor (Righton, Tokyo, Japan) and cycloplegic retinoscopy values were substituted when available.

Based on SE, eyes were categorized as hyperopic (SE ≥ +0.50 diopters [D]), myopic (SE ≤ –0.50 D), or emmetropic (–0.50 D < SE < +0.50 D) [17, 18]. Astigmatism was defined as CYL ≤ –0.50 D [17, 19]. Anisometropia was defined as an interocular difference in SE of ≥ 1.00 D [20]. RE severity was classified by absolute magnitude: mild (0.50–1.75 D), moderate (2.00–3.75 D), moderately severe (4.00–5.75 D), and severe (≥6.00 D) for both myopia and hyperopia; the same thresholds were applied for CYL [17].

### Vision screening protocol

Initial screenings were performed on-site by trained technicians using the Retinomax K-Plus 3 Autorefractor. Referral thresholds included SPH ≥ +1.75 D or SPH ≤ –3.25 D, CYL ≤ –1.50 D, or an interocular difference with ΔSPH ≥ 1.50 D or with ΔCYL ≥ 1.00 D [17, 21, 22]. Children who passed the autorefractor screening but required new spectacles with their current prescription were recorded as ‘fit-for-frames’ (FFF).

### Comprehensive eye examination

Children whose parents provided informed consent received a full examination inside the UCI EyeMobile clinic, performed by one of three licensed pediatric optometrists. Uncorrected visual acuity (UCVA) and best-corrected visual acuity (BCVA) were assessed using Lea symbols, HOTV matching, or Snellen charts, selected according to the child’s age, cooperation, and developmental ability. VA was expressed as the logarithm of the minimum angle of resolution (logMAR), calculated as the negative log of the measured VA score.

Refraction was assessed using the Retinomax autorefractor, manifest phoropter refraction, and retinoscopy. All children underwent non-cycloplegic measurements, with cycloplegic refraction performed when clinically indicated. Cycloplegia was induced with 1% tropicamide with 2.5% phenylephrine administered as a mydriatic to facilitate pupillary dilation; repeat refraction was performed approximately 30 min later. Overall, 2599 of 3350 children (77.6%) underwent cycloplegic refraction, while 751 children (22.4%) completed a non-dilated examination without cycloplegia.

Additional testing included cover testing at distance (20 feet) and near fixation (40 cm), ocular motility assessment, pupillary light reflex, stereoacuity (Titmus test) and color vision (Good-Lite ColorCheck). The anterior segment was evaluated with slit-lamp biomicroscopy and the posterior segment by binocular indirect ophthalmoscopy. Strabismus, when suspected, was confirmed with prism and alternate cover testing. Children with BCVA of 20/40 or worse received updated spectacle prescriptions and new frames at no cost, with counseling on proper use. Those requiring further assessment, such as with the Baylor-Video Acuity Tester (BVAT) or comprehensive ophthalmologic evaluation (Oph), were referred to the UCI Ophthalmology clinic. Referral criteria included suspected amblyopia, strabismus, abnormal red reflex, nystagmus, and other ocular pathology.

### Amblyopia suspect definition and assessment

Amblyopia suspect was defined at the child level as BCVA ≤ 20/40 ( ≥ 0.3 logMAR) in either eye at the time of examination, in the presence of at least one amblyogenic risk factor (ARF), including significant RE, anisometropia, or strabismus [23, 24]. Children with structural ocular pathology that could independently account for reduced VA were excluded. Structural causes were excluded based on slit-lamp biomicroscopy and dilated fundus examination. These included visually significant media opacities (e.g. cataract or corneal opacity), retinal pathology (e.g. macular abnormalities or retinal scarring), optic nerve abnormalities (e.g. optic nerve hypoplasia or pallor), or other ocular conditions deemed by the examining optometrist to explain reduced VA independent of amblyopia.

All refractive data used for amblyopia suspect classification were obtained under cycloplegia; children who did not undergo cycloplegic examination were excluded from amblyopia suspect analyses. Because VA was assessed at a single examination visit without longitudinal follow-up to document response to optical correction, all cases meeting criteria were classified as amblyopia suspect rather than definitive amblyopia, regardless of ARF type.

Unilateral amblyopia suspect additionally required a ≥ 2-line interocular BCVA difference or fixation preference in conjunction with ≥1 ARF. Bilateral amblyopia suspect was defined using age-appropriate BCVA thresholds (>0.40 logMAR for ages 3 to <4 years, >0.30 for 4 to < 5 years, ≥0.20 for ≥5 years) with ≥1 ARF per eye, consistent with the 2022 Amblyopia Preferred Practice Pattern guidelines from the American Academy of Ophthalmology [24]. Age-specific BCVA thresholds were applied exclusively for amblyopia suspect classification. Children meeting criteria were referred for comprehensive ophthalmologic evaluation.

### Geospatial linkage and neighborhood indices

**Fig. 2:**
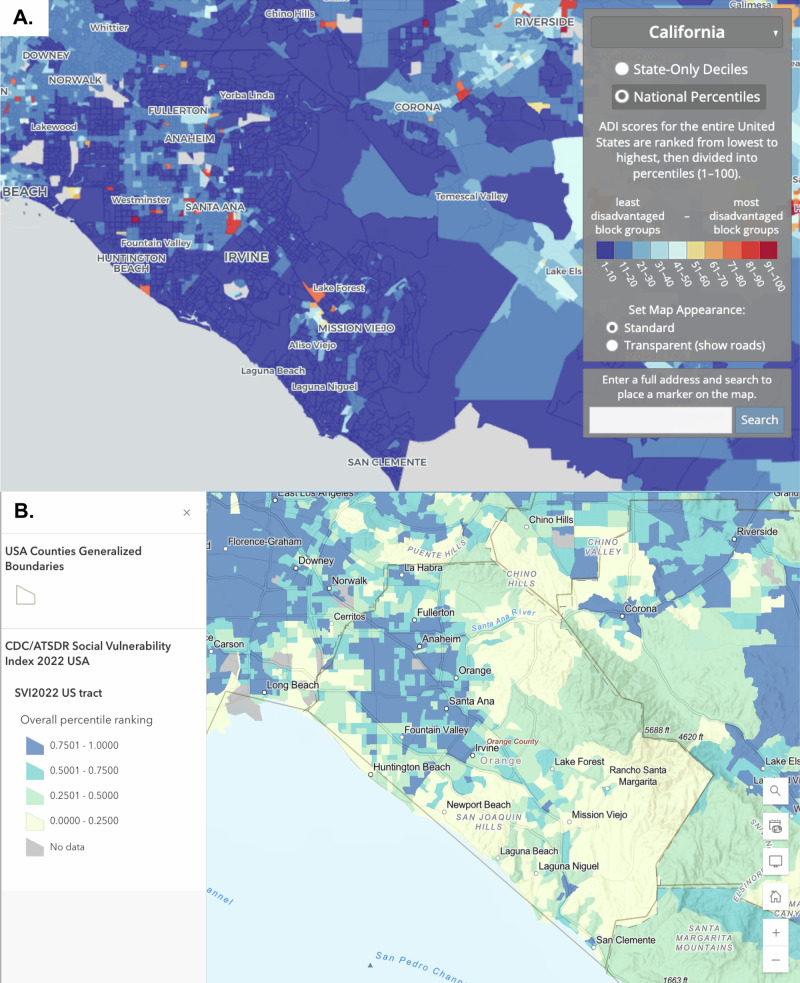
Geographic distribution of neighborhood-level deprivation and social vulnerability in Orange County, California. **A** Area Deprivation Index by census block group, displayed as national percentile rankings from least to most disadvantaged. **B** Social Vulnerability Index by census tract, displayed as overall percentile rankings from lowest to highest vulnerability. Maps adapted from the Neighborhood Atlas (University of Wisconsin School of Medicine and Public Health) and the CDC/ATSDR Social Vulnerability Index interactive mapping tools.

To assess neighborhood-level exposures, we used two validated, nationally standardized indices: the ADI and the SVI, both assigned based on each child’s school address [8, 9, 11, 25]. Addresses were batch-geocoded using the Google Maps Geocoding API and linked to U.S. Census geographic units via the 2022 TIGER/Line shapefiles, yielding census block group identifiers (for ADI) and census tract identifiers (for SVI) used to assign index values [26]. A visual overview of the ADI and SVI distributions in Orange County, California, is provided (Fig. 2).

ADI values were obtained from the 2022 Neighborhood Atlas (version 4.0.1; University of Wisconsin School of Medicine and Public Health), derived from the 2018–2022 American Community Survey (ACS) [11]. ADI is reported at the census block group level as national percentiles from 0 (least deprived) to 100 (most deprived), based on 17 socioeconomic indicators including income, education, employment, and housing quality [11].

SVI values were obtained from the 2022 Centers for Disease Control and Prevention/Agency for Toxic Substances and Disease Registry (CDC/ATSDR) dataset, also derived from the 2018–2022 ACS [10]. SVI is reported at the census tract level as a composite score ranging from 0 to 1, with higher values indicating greater neighborhood vulnerability. SVI incorporates 16 variables grouped into four domains: SES, household composition and disability, minority status and language, and housing type and transportation [10].

For analysis, both ADI and SVI were stratified into four national quartiles (Q1–Q4), with Quartile 1 representing the least deprived or vulnerable neighborhoods and Quartile 4 the most. Complete lists of all 17 ADI indicators and 16 SVI indicators are provided in Supplemental Figs. 1 and 2. Clinical examiners and clinic staff were masked to ADI and SVI status, as neighborhood indices were assigned retrospectively during analysis.

### Statistical analysis

Descriptive statistics summarized demographic characteristics, vision screening and referral outcomes, clinical diagnoses (including RE prevalence and VA distributions), and amblyopia suspect status. Analyses were conducted at both the child and eye levels, depending on the outcome of interest. Screening outcomes, amblyopia suspect classification, and all regression analyses were performed at the child level, whereas VA distributions and RE classifications were descriptively summarized at the eye level. Additionally, descriptive summaries of BCVA distributions are also reported at the eye level, but regression analyses examining poor BCVA as a binary outcome (as defined below) were conducted at the child level. Analyses of poor BCVA and amblyopia suspect were restricted to children who received cycloplegic refraction. Differences in sample sizes across analyses reflect real-world constraints of a school-based mobile clinic setting, including age-appropriate testing protocols, variable cooperation and attention span in young children, time limitations during on-site examinations, and clinical judgment regarding test appropriateness.

Group-level comparisons of categorical variables across ADI and SVI quartiles were assessed using Pearson chi-square tests. When overall chi-square tests were statistically significant, pairwise post-hoc z-tests were performed. Ordered associations were evaluated with Cochran–Armitage trend tests to test for monotonic trends across increasing ADI and SVI quartiles.

The primary regression outcome was poor BCVA, defined at the child level as having either eye worse than 20/30 (logMAR > 0.20). A key secondary outcome was amblyopia suspect (yes/no at the child level). For each outcome, separate multivariable logistic regression models were fit for ADI and SVI (Q4 vs Q1), adjusted for age, sex, race/ethnicity, and RE type and severity. Effect modification by race/ethnicity was assessed using interaction terms (race × ADI and race × SVI). When interactions were significant, stratified multivariable logistic regression models were fit within racial/ethnic groups to estimate adjusted odds of poor BCVA in Q4 compared to Q1, adjusted for age, sex, and RE type and severity. Post-estimation Wald tests using seemingly unrelated estimation (SUEST) were performed to compare interaction effects between ADI- and SVI-based models within racial/ethnic groups.

Model calibration was evaluated using the Hosmer–Lemeshow goodness-of-fit test and discrimination using the area under the receiver operating characteristic curve (AUC). ADI and SVI were modeled in separate regression frameworks because they capture overlapping socioeconomic constructs; joint inclusion was avoided to minimize multicollinearity and preserve interpretability of index-specific associations. All statistical tests were two-sided, with *p* < 0.05 considered significant. Analyses were performed using STATA 17.0 (StataCorp LLC, College Station, TX, USA).

Given the evaluation of multiple related outcomes, analyses were conducted in an exploratory framework. Poor BCVA was prespecified as the primary outcome due to its clinical relevance, with amblyopia suspect designated as a key secondary outcome. Other screening and RE analyses were descriptive and hypothesis-generating.

## Results

### Screening cohort and demographics

Between 2021 and 2024, a total of 15,842 children were screened through the UCI EyeMobile program. Of these, 11,199 (70.7%) passed initial vision screening. The remaining 4643 children (29.3%) did not pass and were triaged based on clinical findings. Of this group, 3609 (22.8% of total screened) met referral criteria and were referred for comprehensive ophthalmologic evaluation, 825 (5.2%) met criteria for FFF and were provided new spectacles on the same day, 59 (0.4%) passed upon retesting with their previously prescribed EyeMobile spectacles, and 150 (0.9%) were not screenable due to age, cooperation, or testing limitations. Complete screening outcomes are summarized in Table 1.

**Table 1:** Demographics and vision screening outcomes for 15,842 children stratified by quartiles of Area Deprivation Index and Social Vulnerability Index.

Variable	Total (*n* = 15,842)	ADI Q1 (*n* = 2420)	ADI Q2 (*n* = 3485)	ADI Q3 (*n* = 4180)	ADI Q4 (*n* = 5757)	SVI Q1 (*n* = 2800)	SVI Q2 (*n* = 3700)	SVI Q3 (*n* = 4470)	SVI Q4 (*n* = 4872)
**Ethnicity**									
Hispanic	8193 (51.7%)	1067 (44.1%)	1945 (55.8%)	1988 (47.6%)	3193 (55.4%)	924 (33.0%)	1688 (45.6%)	2520 (56.4%)	3061 (62.8%)
Caucasian	3718 (23.5%)	814 (33.6%)	839 (24.1%)	1030 (24.6%)	1035 (18.0%)	1122 (40.1%)	1025 (27.7%)	874 (19.6%)	697 (14.3%)
Asian	2650 (16.7%)	397 (16.4%)	398 (11.4%)	901 (21.6%)	954 (16.6%)	605 (21.6%)	687 (18.6%)	691 (15.5%)	667 (13.7%)
African American	394 (2.5%)	35 (1.5%)	65 (1.9%)	59 (1.4%)	235 (4.1%)	33 (1.2%)	65 (1.8%)	105 (2.3%)	191 (3.9%)
Middle Eastern	887 (5.6%)	107 (4.4%)	238 (6.8%)	202 (4.8%)	340 (5.9%)	116 (4.1%)	235 (6.4%)	280 (6.3%)	256 (5.3%)
**Sex **									
Male	8025 (50.7%)	1234 (51.0%)	1771 (50.8%)	2134 (51.0%)	2886 (50.1%)	1400 (50.0%)	1881 (50.8%)	2258 (50.5%)	2486 (51.0%)
Female	7817 (49.3%)	1186 (49.0%)	1714 (49.2%)	2046 (49.0%)	2871 (49.9%)	1400 (50.0%)	1819 (49.2%)	2212 (49.5%)	2386 (49.0%)
**Total screened**									
Screen pass	11,199 (70.7%)	1889 (78.0%)	2750 (78.9%)	2725 (65.2%)	3835 (66.7%)	2099 (75.0%)	2730 (73.8%)	3180 (71.2%)	3190 (65.5%)
Referred	3609 (22.8%)	397 (16.4%)	525 (15.1%)	1166 (27.9%)	1521 (26.4%)	504 (18.0%)	798 (21.6%)	1104 (24.7%)	1203 (24.7%)
Unable to screen	150 (0.9%)	13 (0.6%)	28 (0.8%)	45 (1.1%)	64 (1.1%)	25 (0.9%)	33 (0.9%)	46 (1.0%)	46 (0.9%)
Fit-for-frames	825 (5.2%)	117 (4.8%)	180 (5.1%)	219 (5.2%)	309 (5.3%)	158 (5.6%)	125 (3.4%)	129 (2.9%)	413 (8.5%)
Previously dispensed glasses	59 (0.4%)	4 (0.2%)	2 (0.1%)	25 (0.6%)	28 (0.5%)	14 (0.5%)	14 (0.4%)	11 (0.2%)	20 (0.4%)

‘Referred’ indicates referral for further comprehensive eye examination after failed screening. ‘Fit-for-Frames’ are children who passed the autorefractor screening but required new spectacles and received them on the same day of the visit. ‘Previously Dispensed Glasses’ are students presenting with EyeMobile-provided spectacles from prior visits who retested using them and passed.

Values are reported as a number (%). Quartiles: Q1 = least deprived/vulnerable, Q4 = most deprived/vulnerable. *P*-values calculated by chi-square test for association across quartiles. Race/ethnicity distributions differed significantly across both ADI and SVI quartiles (overall chi-square *p* < 0.001 for each), with Hispanic children disproportionately represented in Q4 and Caucasian children more frequently represented in Q1. Vision screening outcomes (screen pass, referral, unable to screen, fit-for-frames, previously dispensed glasses) also differed significantly across ADI and SVI quartiles (overall chi-square *p* < 0.001 for each). Sex distribution did not differ significantly across quartiles (*p* > 0.05).

A total of 3885 children presented for comprehensive ophthalmologic evaluation, including the 3609 children originally referred following initial screening and an additional 276 children who independently scheduled follow-up visits. Of these 3885 children, 3350 (86.3%) completed a full comprehensive eye examination, 513 (13.2%) did not show or did not provide consent, and 22 (0.6%) were found to already be under the care of an outside medical provider and were therefore not eligible for further examination. The latter two groups were excluded from downstream clinical analyses, yielding a final analytic cohort of 3350 children (Fig. 1). Clinical examination outcomes are summarized in Supplemental Table 1.

Among the screened children, the cohort was 50.7% male and 49.3% female. Hispanic children represented the largest racial/ethnic group (51.7%), followed by Caucasian (23.5%), Asian (16.7%), Middle Eastern (5.6%) and African American (2.5%) children. The distribution of race and ethnicity varied significantly across both ADI and SVI quartiles (*p* < 0.001), with Hispanic children concentrated in Q4 (ADI: 55.4%, SVI: 62.8%) and Caucasian children more frequently represented in Q1 (ADI: 33.6%, SVI: 40.1%).

Baseline demographic characteristics as well as the distribution of ADI and SVI quartiles were similar between children who were initially screened and those who ultimately underwent comprehensive examination (Supplemental Table 2).

### Visual acuity outcomes

VA distributions are shown in Supplemental Table 3. For uncorrected distance vision, among 4786 eyes tested, 55.3% achieved 20/30 or better (<0.2 logMAR), 24.6% were 20/40–20/50 (0.3–0.4 logMAR), 11.6% between 20/63 and 20/80 (0.5–0.6 logMAR), and 8.6% had vision 20/100 or worse (>0.7 logMAR). For near vision, among 4,873 eyes, 52.2% achieved 20/30 or better and 10.9% worse than 20/100. After correction with spectacles, BCVA improved substantially: among 2431 eyes, 84.0% achieved 20/30 or better, 12.3% were 20/40–20/50, 2.4% between 20/63 and 20/80, and only 1.3% remained 20/100 or worse.

### Refractive error outcomes

Among 5384 eyes with complete RE data (Supplemental Table 4), 78% were measured using autorefractor values and 22% using retinoscopy. Overall, 36.9% of eyes were emmetropic, 25.8% myopic, and 37.3% hyperopic. Most myopia was mild (0.50–1.99 D; 74.7%) and 88.7% of myopic eyes had concurrent astigmatism. Similarly, most hyperopia was mild (71.0%), with 77.7% associated with astigmatism. Overall, 73.6% of eyes had astigmatism, most commonly mild (59.5%). Anisometropia was present in 9.1% of eyes with paired refraction data (246 eyes of 2,692), of which 82.5% were spherical, 37.0% cylindrical, and 89.0% concurrent with astigmatism.

Chi-square testing showed significant variation in RE prevalence across both ADI and SVI quartiles. Myopia was significantly less common in Q4 (ADI Q1: 29.1% vs. Q4: 20.5%; SVI Q1: 32.0% vs. Q4: 18.2%, *p* < 0.001). Hyperopia did not differ (*p* = 0.23). Astigmatism was significantly more common in higher quartiles (ADI Q1: 15.0% vs. Q4: 36.0%; SVI Q1: 19.0% vs. Q4: 31.0%; *p* < 0.01). Emmetropia was lower in Q4 (ADI Q1: 40.0% vs. Q4: 32.4%; SVI Q1: 40.0% vs. Q4: 33.7%; *p* < 0.01). Anisometropia showed no significant variation between quartiles (*p* = 0.18).

### BCVA by race, ADI and SVI

Eye-level distributions of poor BCVA (BCVA > 0.2 logMAR, worse than 20/30 in either eye) after optical correction are summarized in Table 2. Across increasing ADI quartiles, significant differences in the proportion of eyes with poor BCVA were observed among Hispanic (*p* < 0.001), Caucasian (*p* = 0.001) and Asian eyes (*p* = 0.021). Post-hoc pairwise comparisons showed that Hispanic eyes in ADI Q4 had significantly higher rates of poor BCVA compared to Q1 (adjusted *p* < 0.001), with a similar pattern observed among Asian eyes (adjusted *p* = 0.01). No significant differences were observed for Caucasian eyes (*p* = 0.64).

**Table 2:** Racial and ethnic disparities in best-corrected visual acuity worse than 20/30 (logMAR > 0.2).

Race/ Ethnicity	ADI Q1 (*n* = 25)	ADI Q2 (*n* = 20)	ADI Q3 (*n* = 115)	ADI Q4 (*n* = 228)	Total Eyes (*n* = 388)	*p* (chi-square)	*p* (trend)	SVI Q1 (*n* = 48)	SVI Q2 (*n* = 72)	SVI Q3 (*n* = 114)	SVI Q4 (*n* = 154)	Total Eyes (*n* = 388)	*p* (chi-square)	*p* (trend)
Hispanic	8 (32.0%)	7 (35.0%)	60 (52.2%)	145 (63.6%)	220 (56.7%)	<0.001	<0.001	18 (37.5%)	30 (41.7%)	67 (58.8%)	91 (59.1%)	206 (53.1%)	<0.001	<0.001
Caucasian	7 (28.0%)	5 (25.0%)	25 (21.7%)	45 (19.7%)	82 (21.1%)	0.001	0.004	15 (31.3%)	20 (27.8%)	22 (19.3%)	25 (16.2%)	82 (21.1%)	0.002	0.005
Asian	4 (16.0%)	3 (15.0%)	16 (13.9%)	22 (9.7%)	45 (11.6%)	0.021	0.038	6 (12.5%)	8 (11.1%)	11 (9.6%)	16 (10.4%)	41 (10.6%)	0.015	0.031
African American	3 (12.0%)	3 (15.0%)	8 (7.0%)	10 (4.4%)	24 (6.2%)	0.217	0.111	4 (8.3%)	7 (9.7%)	6 (5.3%)	13 (8.4%)	30 (7.7%)	0.190	0.105
Middle Eastern	3 (12.0%)	2 (10.0%)	6 (5.2%)	6 (2.6%)	17 (4.4%)	0.307	0.167	5 (10.4%)	7 (9.7%)	8 (7.0%)	9 (5.8%)	29 (7.5%)	0.312	0.171

This table presents the distribution of eyes with best-corrected visual acuity (BCVA) worse than 20/30 across quartiles of the Area Deprivation Index (ADI) and Social Vulnerability Index (SVI), stratified by race and ethnicity. Each row represents a racial or ethnic group, with values reported as the number and percentage of affected eyes within each ADI and SVI quartile. Analyses were conducted at the eye level among eyes with available best-corrected visual acuity measurements (total eyes with poor BCVA = 388). Chi-square tests were used to assess overall differences across quartiles and Cochran–Armitage trend tests were used to evaluate directional trends.

Similar patterns were observed across SVI quartiles, with significant differences among Hispanic (*p* < 0.001), Caucasian (*p* = 0.002) and Asian eyes (*p* = 0.015). Post-hoc analyses confirmed higher rates among Hispanic eyes in Q4 vs Q1 (adjusted *p* < 0.001) and among Asian eyes in Q4 (adjusted *p* < 0.01), with no differences for Caucasian eyes (*p* = 0.45).

Cochran–Armitage trend tests confirmed significant monotonic increases in the proportion of eyes with poor BCVA across increasing ADI quartiles for Hispanic (*p* < 0.001), Asian (*p* = 0.038) and Caucasian eyes (*p* = 0.004). Across SVI quartiles, significant upward trends were observed for Hispanic (*p* < 0.001) and Caucasian eyes (*p* = 0.005).

### Multivariable regression

Multivariable logistic regression confirmed that ADI and SVI, when modeled separately, were independently associated with poor BCVA, along with race/ethnicity and sex (Supplemental Table 5). After adjustment, children in ADI Q4 had 51% higher odds of poor BCVA compared to Q1 (aOR 1.51, 95% CI 1.13–2.01, *p* = 0.006), while those in SVI Q4 had 61% higher odds (aOR 1.61, 95% CI 1.18–2.20, *p* = 0.002). Hispanic ethnicity was associated with 73% higher odds of poor BCVA compared to Caucasian children (aOR 1.73, 95% CI 1.22–2.41, *p* = 0.002). Asians had elevated odds that did not reach statistical significance (aOR 1.35, 95% CI 0.91–1.71, *p* = 0.24). Females had slightly higher odds than males (aOR 1.25, 95% CI 1.01–1.55, *p* = 0.041). Both ADI and SVI models had very good discrimination (AUC = 0.826; 0.854) and acceptable calibration (Hosmer–Lemeshow *p* = 0.41; 0.48).

**Fig. 3:**
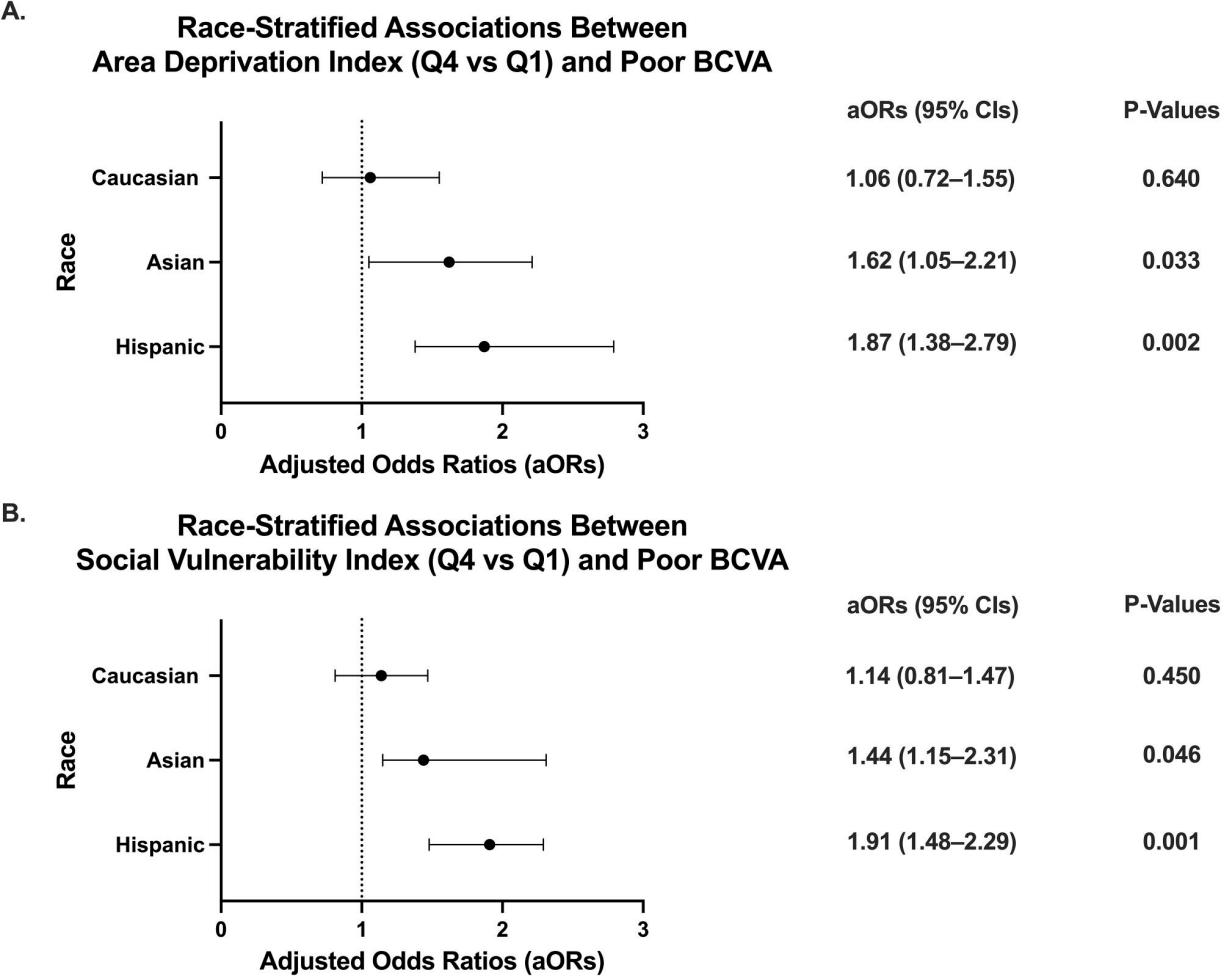
Race-stratified associations of neighborhood deprivation and social vulnerability with poor best-corrected visual acuity. **A** Adjusted odds ratios for poor best-corrected visual acuity comparing the highest versus lowest quartiles (Q4 vs Q1) of the Area Deprivation Index, stratified by race. **B** Adjusted odds ratios for poor best-corrected visual acuity comparing the highest versus lowest quartiles (Q4 vs Q1) of the Social Vulnerability Index, stratified by race. All estimates are shown with 95% confidence intervals.

Interaction analyses showed significant race × ADI (*p* < 0.01) and race × SVI (*p* = 0.02) effects, indicating that the effects of neighborhood deprivation and vulnerability varied by race/ethnicity. Stratified models by race/ethnicity demonstrated that Hispanic children in ADI Q4 had almost two-fold higher odds of poor BCVA compared to Hispanic children in Q1 (aOR 1.87, 95% CI 1.38–2.79, *p* = 0.002). Asian children showed a similar though smaller effect (aOR 1.62, 95% CI 1.05–2.21, *p* = 0.033). No significant quartile-based difference was observed among Caucasian children (aOR 1.06, 95% CI 0.72–1.55, *p* = 0.64). Comparable results were seen using SVI quartiles, with significant associations for Hispanic (aOR 1.91, 95% CI 1.48–2.29, *p* = 0.001) and Asian children (aOR 1.44, 95% CI 1.15–2.31, *p* = 0.046) in Q4 vs Q1, but not Caucasian children (aOR 1.14, 95% CI 0.81–1.47, *p* = 0.45) (Fig. 3). In post-estimation comparisons, SUEST-based Wald tests demonstrated that the association between neighborhood context and poor BCVA was stronger for SVI than ADI among Hispanic children (*p* = 0.038), while no significant differences between indices were observed for Asian (*p* = 0.426) or Caucasian children (*p* = 0.749).

### Amblyopia suspect outcomes

Among 3350 children examined, 334 (10.0%) met criteria for amblyopia suspect based on reduced BCVA in the presence of ARFs (Supplemental Table 6). Rates differed significantly across ADI and SVI quartiles (*p* < 0.01). Children in ADI Q4 had a higher prevalence than Q1 (12.4% vs 7.2%, *p* < 0.01), with similar differences for SVI (12.4% vs 7.5%, *p* < 0.01). Prevalence rose monotonically with increasing disadvantage (*p*-trend < 0.001 for both indices). In multivariable logistic regression, amblyopia suspect was more likely among children in ADI Q4 vs Q1 (aOR 1.78, 95% CI 1.30–2.43, *p* < 0.001) and in SVI Q4 vs Q1 (aOR 1.82, 95% CI 1.33–2.48, *p* < 0.001).

## Discussion

In our community-based cohort of 15,842 children screened through the UCI EyeMobile program, neighborhood disadvantage measured by ADI and SVI was consistently associated with pediatric vision outcomes. Children attending schools in ADI/SVI Q4 neighborhoods had higher referral rates and were more likely to require new spectacles despite passing initial screening, highlighting unmet refractive needs identified only through comprehensive examination.

RE patterns varied by neighborhood context. Children in ADI/SVI Q4 had a lower myopia prevalence but higher astigmatism prevalence, while hyperopia and anisometropia did not differ across quartiles. Myopia prevalence was significantly lower in the most disadvantaged quartiles (ADI: 29.1% in Q1 vs 20.5% in Q4; SVI: 32.0% vs 18.2%), consistent with prior studies reporting higher myopia prevalence in more affluent and urban populations, potentially related to differences in educational and visual demands [27–29]. Conversely, astigmatism prevalence was significantly higher (ADI: 15.0% vs 36.0%; SVI: 19.0% vs 31.0%) in disadvantaged quartiles, likely reflecting differences in population composition and access to timely refractive assessment rather than a direct causal effect of deprivation, as astigmatism varies by race/ethnicity, age and patterns of detection [30–32]. Longitudinal studies with individual- and household-level data will be required to distinguish compositional from contextual contributions to astigmatism risk.

VA outcomes showed similar patterns. At the eye level, poor BCVA increased monotonically across ADI and SVI quartiles (ADI: 16.3% in Q1 to 18.1% in Q4; SVI: 15.5% to 19.9%). At the child level, amblyopia suspects showed larger absolute differences (ADI: 7.2% to 12.4%; SVI: 7.5% to 12.4%), consistent with prior literature documenting delayed detection and treatment in socioeconomically disadvantaged populations [33, 34]. Although mechanisms could not be directly assessed in this study, such disparities may reflect barriers to early screening, timely spectacle wear, and ophthalmologic follow-up, warranting further investigation. Incorporating amblyopia suspect outcomes extends our findings by suggesting that neighborhood disadvantage is associated not only with RE burden and BCVA, but also with markers of increased risk for adverse visual development. In the context of large school-based screening programs, even modest increases in poor BCVA at the eye level may translate into substantial numbers of children at potential risk for persistent visual impairment, underscoring the clinical relevance of these findings.

Consistent with our hypothesis, higher neighborhood disadvantage measured by either ADI or SVI was associated with poorer pediatric vision outcomes, supporting the relevance of shared socioeconomic dimensions captured by both indices. We further hypothesized that SVI would demonstrate differential associations within racial and ethnic subgroups, a hypothesis supported by our race-stratified analyses in which SVI demonstrated stronger neighborhood gradients in poor BCVA among Hispanic children. Post-estimation Wald tests confirmed that the association between neighborhood context and poor BCVA was significantly stronger for SVI than ADI among Hispanic children, while no index-specific differences were observed among Asian or Caucasian children.

This pattern aligns with SVI’s incorporation of minority status and language vulnerability domains, which may be particularly salient in our predominantly Spanish-speaking Hispanic population and better capture barriers related to language discordance, healthcare navigation, and continuity of follow-up care. These results indicate that while both models demonstrated similar discrimination (AUCs 0.826 and 0.854), SVI provided further contextual insight in specific populations, therefore complementing rather than duplicating the information captured by ADI. Similar dual-index approaches have been used in pediatric and adult disparities research to distinguish socioeconomic deprivation from broader social vulnerability [14, 15].

The most novel finding was the significant interaction between race/ethnicity and ADI/SVI, demonstrating that neighborhood context and race/ethnicity jointly shape vision outcomes. ADI and SVI remained independently associated with poor BCVA after adjustment for age, sex, race/ethnicity and RE type/severity, indicating that neighborhood disadvantage contributes to risk beyond individual demographic and clinical characteristics. However, neighborhood disadvantage did not affect all groups uniformly, with more substantial effects and modifications within some racial/ethnic groups. Hispanic and Asian children attending schools in Q4 neighborhoods had significantly higher odds of poor BCVA compared with same-ethnicity peers in Q1, whereas no gradient was observed among Caucasian children (Fig. 3). These results highlight that inequities may exist not only between racial and ethnic groups but also within them, consistent with intersectional perspectives suggesting that structural context differentially shapes risk across subgroups [35].

Among Hispanic and Asian children, the amplified neighborhood gradients may reflect converging structural and social barriers affecting access to and continuity of pediatric eye care. Prior studies have shown that language discordance, challenges navigating the healthcare system, and immigration-related concerns can influence healthcare utilization and follow-up among minority and immigrant populations, even when services are available [36–38]. In Orange County, many Hispanic and Asian families are immigrants or first-generation Americans; such contextual factors may plausibly shape care-seeking behaviors and continuity of vision care beyond initial school-based screening, but further investigation is needed [38–40].

From a practice standpoint, these findings support targeted approaches to improve screening-to-treatment continuity in high-ADI/SVI communities. Similar to the UCI EyeMobile program, the Vision for Baltimore program demonstrated that school-based screenings, free on-site glasses distribution, and support from community health workers improved spectacle adherence and academic performance [41, 42]. Strategies to strengthen continuity can include extended clinic hours, tele-ophthalmology, and mobile follow-up models that reduce transportation and scheduling barriers [43, 44]. Pediatric ophthalmologists are uniquely positioned to identify children from high-ADI/SVI neighborhoods through early school-based screening programs. Beyond vision care, they can help facilitate access to nutritional assistance, housing resources, and developmental resources, often in coordination with school nurses and social workers.

From a policy standpoint, ADI and SVI can guide health equity planning and quality improvement. Health systems have used ADI for value-based reimbursement and hospital readmission risk adjustment [45, 46]. Our findings advocate for a similar role in pediatric vision care. State Medicaid and Children’s Health Insurance Programs could fund school-based clinics, integrate eye care into federally qualified health centers, and deliver multilingual education in the highest ADI/SVI quartiles.

### Strengths, limitations, and future directions

While this study provides novel insight into neighborhood-level inequities in pediatric vision outcomes, several limitations merit consideration. ADI and SVI values were assigned using school addresses rather than household addresses, as residential address data were unavailable under the UCI EyeMobile program’s IRB protocol and data-sharing agreements with schools. Consequently, neighborhood exposure reflects the school-community context rather than precise household-level socioeconomic conditions. Although public school attendance in Orange County is largely determined by geographically defined attendance zones, school-based geocoding may not fully capture within-neighborhood heterogeneity or individual residential circumstances.

Further, only a subset of screened children completed comprehensive eye examinations, which may limit generalizability. Although baseline demographic characteristics and ADI/SVI quartile distributions were similar between screened children and those who underwent examination, unmeasured factors such as parental engagement, availability for follow-up, or healthcare access may influence examination completion and contribute to residual selection bias. Additionally, not all children completed every examination component due to age, cooperation, or clinical appropriateness, resulting in variable denominators across VA and RE analyses. Such missingness may be non-random, warranting careful interpretation of estimates.

Several methodological considerations related to outcome assessment and modelling should also be noted. Cycloplegic refraction was performed using 1% tropicamide, a relatively mild cycloplegic regimen commonly used in school-based and mobile eye care settings. As a result, latent hyperopia may have been underestimated in some children, with implications for RE characterization and amblyopia suspect classification. Amblyopia outcomes should be interpreted as amblyopia suspect, as VA was assessed at a single visit without long-term follow-up to confirm persistence after optical correction, reflecting the constraints of a mobile clinic setting. Although multivariable models adjusted for age, sex, race/ethnicity and RE type and severity, residual confounding from unmeasured individual-level factors, including household socioeconomic characteristics, insurance status, or primary language, remains possible. Prior spectacle use was not included in primary models because it reflects prior healthcare utilization rather than baseline visual status and could potentially obscure associations between neighborhood context and outcomes. Children were analyzed as independent observations, although students attending the same school may share unmeasured contextual characteristics beyond those captured by ADI and SVI, potentially underestimating standard errors. Future studies may address this using multilevel modelling or cluster-robust variance estimation. Finally, the cross-sectional design precludes causal inference, and African American and Middle Eastern children were underrepresented, limiting the precision of subgroup estimates.

Despite these limitations, this study represents the first large-scale U.S. application of ADI and SVI to pediatric vision screening and examination outcomes in a racially diverse, predominantly underserved population. Standardised BCVA assessment, detailed RE characterization, linkage to validated neighborhood indices and formal interaction analyses strengthen the study’s findings. Future longitudinal studies incorporating household-level socioeconomic data, stronger cycloplegic protocols when feasible, and extended follow-up will be essential to clarify mechanisms and inform equity-focused pediatric vision interventions.

## Summary

### What is known about this topic

- Neighborhood disadvantage affects pediatric health outcomes, but its relationship with childhood vision disorders remains underexplored. Previous studies have linked higher Area Deprivation Index and Social Vulnerability Index scores to reduced well-child visits, lower vaccination rates, and increased obesity risk in children, highlighting broader health disparities.

### What this study adds

- This is the first large-scale U.S. study to apply the Area Deprivation Index and Social Vulnerability Index to vision screening outcomes. Neighborhood disadvantage, both independently and through interaction with race and ethnicity, was associated with poorer visual acuity and greater amblyopia suspect risk, underscoring the need for equity-focused screening strategies.

## Supplementary information

Supplementary Material

## Data Availability

The dataset includes protected and deidentified patient health information collected under HIPAA-compliant protocols and participant-informed consent. Per IRB and institutional policy, this data will not be publicly available.

## References

[1] Chen AM, Cotter SA. The amblyopia treatment studies: implications for clinical practice. Adv Ophthalmol Optom. 2016;1:287–305. 28435934 10.1016/j.yaoo.2016.03.007 PMC5396957

[2] Blair K, Cibis G, Zeppieri M, Gulani AC Amblyopia. In: StatPearls. Treasure Island: StatPearls Publishing; 2025. Available at: http://www.ncbi.nlm.nih.gov/books/NBK430890/.

[3] Anon. Vision Problems | Boston Children’s Hospital. Available at: https://www.childrenshospital.org/conditions-treatments/vision-problems.

[4] Kim H, Shahraki K, Suh DW. Myopia trends among children and adolescents: a nationwide study in South Korea. J Am Assoc Pediatr Ophthalmol Strabismus. 2024;28. Available at: https://www.jaapos.org/article/S1091-8531(24)00249-0/abstract. 10.1016/j.jaapos.2024.103969 38997085

[5] Antonio-Aguirre B, Ambrosino CM, Dai X, Collins ME. Addressing health disparities in pediatric eye care for school-age children: a call to action. Transl Vis Sci Technol. 2023;12:17. 37962540 10.1167/tvst.12.11.17 PMC10653256

[6] Acevedo-Garcia D, Noelke C, McArdle N, Sofer N, Hardy EF, Weiner M, et al. Racial and ethnic inequities in children’s neighborhoods: evidence from the new child opportunity index 2.0. Health Aff. 2020;39:1693–701. 10.1377/hlthaff.2020.00735 33017244

[7] Diez Roux AV, Mair C. Neighborhoods and health. Ann N Y Acad Sci. 2010;1186:125–45. 20201871 10.1111/j.1749-6632.2009.05333.x

[8] Powell WR, Sheehy AM, Kind AJH. The area deprivation index is the most scientifically validated social exposome tool available for policies advancing health equity. Available at: https://www.healthaffairs.org/do/10.1377/forefront.20230714.676093/full/.

[9] Ng CD, Zhang P, Kowal S. Validating the social vulnerability index for alternative geographies in the United States to explore trends in social determinants of health over time and geographic location. Front Public Health. 2025;13:1547946. 40104116 10.3389/fpubh.2025.1547946 PMC11915720

[10] CDC. Social vulnerability index. Place and health—Geospatial Research, Analysis, and Services Program (GRASP). 2024. Available at: https://www.atsdr.cdc.gov/place-health/php/svi/index.html.

[11] Kind AJH, Buckingham WR. Making neighborhood-disadvantage metrics accessible—the neighborhood atlas. N Engl J Med. 2018;378:2456–8. 29949490 10.1056/NEJMp1802313 PMC6051533

[12] Shaheen A, Medeiros FA, Swaminathan SS. Association between greater social vulnerability and delayed glaucoma surgery. Am J Ophthalmol. 2024;268:123–35. 39089357 10.1016/j.ajo.2024.07.019 PMC11606798

[13] Almidani L, Bradley C, Herbert P, Ramulu P, Yohannan J. The impact of social vulnerability on structural and functional glaucoma severity, worsening, and variability. Ophthalmol Glaucoma. 2024;7:380–90. 38636704 10.1016/j.ogla.2024.03.008 PMC12980543

[14] Jawad K, Feygin YB, Stevenson M, Wattles BA, Porter J, Jones VF, et al. The association between four neighborhood disadvantage indices and child chronic health classifications. Pediatr Res. 2025;99:292–300. 10.1038/s41390-025-04143-5 PMC12920081 40425849

[15] Zolotor A, Huang RW, Bhavsar NA, Cholera R. Comparing social disadvantage indices in pediatric populations. Pediatrics. 2024;154:e2023064463. 39143925 10.1542/peds.2023-064463 PMC11350100

[16] Aris IM, Perng W, Dabelea D, Padula AM, Alshawabkeh A, Vélez-Vega CM, et al. Associations of neighborhood opportunity and social vulnerability with trajectories of childhood body mass index and obesity among US children. JAMA Netw Open. 2022;5:e2247957. 36547983 10.1001/jamanetworkopen.2022.47957 PMC9857328

[17] Hendler K, Mehravaran S, Lu X, Brown SI, Mondino BJ, Coleman AL. Refractive Errors and Amblyopia in the UCLA Preschool Vision Program: First-Year Results. Am J Ophthalmol. 2016;172:80–6. 27640004 10.1016/j.ajo.2016.09.010

[18] Flitcroft DI, He M, Jonas JB, Jong M, Naidoo K, Ohno-Matsui K, et al. IMI—defining and classifying myopia: a proposed set of standards for clinical and epidemiologic studies. Investig Ophthalmol Vis Sci. 2019;60:M20–M30. 30817826 10.1167/iovs.18-25957 PMC6735818

[19] Villegas EA, Alcón E, Artal P. Minimum amount of astigmatism that should be corrected. J Cataract Refract Surg. 2014;40:13–19. 24355718 10.1016/j.jcrs.2013.09.010

[20] Deng L, Gwiazda JE. Anisometropia in children from infancy to 15 years. Investig Ophthalmol Vis Sci. 2012;53:3782. 22589429 10.1167/iovs.11-8727 PMC3390183

[21] Hunter SC, He J, Han M, Suh DW. The UCI eyemobile preschool vision screening program: refractive error and amblyopia results from the 2019–2020 school year. Clin Ophthalmol. 2022;16:4249–55. 36573233 10.2147/OPTH.S382899 PMC9789699

[22] Margines JB, Huang C, Young A, Mehravaran S, Yu F, Mondino BJ, et al. Refractive errors and amblyopia among children screened by the UCLA Preschool Vision Program in Los Angeles County. Am J Ophthalmol. 2020;210:78–85. 31647932 10.1016/j.ajo.2019.10.013

[23] Xiao O, Morgan IG, Ellwein LB, He M. Refractive error study in children study group. Prevalence of amblyopia in school-aged children and variations by age, gender, and ethnicity in a multi-country refractive error study. Ophthalmology. 2015;122:1924–31. 26278861 10.1016/j.ophtha.2015.05.034 PMC6029943

[24] Cruz OA, Repka MX, Hercinovic A, Cotter SA, Lambert SR, Hutchinson AK, et al. Amblyopia preferred practice pattern. Ophthalmology. 2023;130:P136–P178. 36526450 10.1016/j.ophtha.2022.11.003 PMC10701408

[25] Anon. Centers for Disease Control and Prevention Social Vulnerability Index (CDC SVI) validity and reliability assessment. Available at: https://www.atsdr.cdc.gov/place-health/media/pdf/Validation-WhitePaper-508.pdf.

[26] Bureau UC. TIGER/Line Shapefiles. Census.gov. Available at: https://www.census.gov/geographies/mapping-files/time-series/geo/tiger-line-file.html.

[27] Zong Z, Zhang Y, Qiao J, Tian Y, Xu S. The association between screen time exposure and myopia in children and adolescents: a meta-analysis. BMC Public Health. 2024;24:1625. 38890613 10.1186/s12889-024-19113-5 PMC11186094

[28] Rudnicka AR, Kapetanakis VV, Wathern AK, Logan NS, Gilmartin B, Whincup PH, et al. Global variations and time trends in the prevalence of childhood myopia, a systematic review and quantitative meta-analysis: implications for aetiology and early prevention. Br J Ophthalmol. 2016;100:882–90. 26802174 10.1136/bjophthalmol-2015-307724 PMC4941141

[29] Ma Y, Lin S, Li L, Jia Y, Zou H. Socioeconomic mechanisms of myopia boom in China: a nationwide cross-sectional study. BMJ Open. 2021;11:e044608. 34135035 10.1136/bmjopen-2020-044608 PMC8211073

[30] Wen G, Tarczy-Hornoch K, McKean-Cowdin R, Cotter SA, Borchert M, Lin J, et al. Prevalence of myopia, hyperopia, and astigmatism in non-Hispanic white and Asian children: multi-ethnic pediatric eye disease study. Ophthalmology. 2013;120:2109–16. 23953098 10.1016/j.ophtha.2013.06.039 PMC3902090

[31] Tarczy-Hornoch K, Varma R, Cotter SA, McKean-Cowdin R, Lin JH, Borchert MS, et al. Risk factors for astigmatism in preschool children: the multi-ethnic pediatric eye disease and Baltimore pediatric eye disease studies. Ophthalmology. 2011;118:1974–81. 21856010 10.1016/j.ophtha.2011.06.031 PMC3186875

[32] Kulp MT, Vision in Preschoolers (VIP) Study G. Findings from the vision in preschoolers (VIP) study. Optom Vis Sci. 2009;86:619. 19417714 10.1097/OPX.0b013e3181a59bf5 PMC2806243

[33] Repka MX, Li C, Lum F. Multivariable analyses of amblyopia treatment outcomes from a clinical data registry. Ophthalmology. 2023;130:164–6. 36100075 10.1016/j.ophtha.2022.09.005

[34] Yoo SH, Archer DKL, Zickafoose JS, Padovani-Claudio DA. Addressing inequities in amblyopia treatment outcomes. Pediatrics. 2025;155:e2024069410. 10.1542/peds.2024-069410 39914447

[35] Homan P, Brown TH, King B. Structural intersectionality as a new direction for health disparities research. J Health Soc Behav. 2021;62:350–70. 34355603 10.1177/00221465211032947 PMC8628816

[36] Mahajan S, Caraballo C, Lu Y, Valero-Elizondo J, Massey D, Annapureddy AR, et al. Trends in differences in health status and health care access and affordability by race and ethnicity in the United States, 1999–2018. JAMA. 2021;326:637–48. 34402830 10.1001/jama.2021.9907 PMC8371573

[37] Shah NS, Kandula NR, Commodore-Mensah Y, Morey BN, Patel SA, Wong S, et al. Social determinants of cardiovascular health in asian americans: a scientific statement from the American Heart Association. Circulation. 2024;150:e296–e315. 39279648 10.1161/CIR.0000000000001278

[38] Rodríguez RM, Torres JR, Sun J, Alter H, Ornelas C, Cruz M, et al. Declared the impact of the US President’s statements and campaign statements on Latino populations’ perceptions of safety and emergency care access. PLoS ONE. 2019;14:e0222837. 31665147 10.1371/journal.pone.0222837 PMC6821049

[39] Tarraf W, Miranda PY, González HM. Medical expenditures among immigrant and non-immigrant groups in the U.S.: findings from the Medical Expenditures Panel Survey (2000–2008). Med Care. 2012;50:233–42. 22222383 10.1097/MLR.0b013e318241e5c2 PMC3279567

[40] Anon. It’s not you, it’s your heritage | School of Social Ecology. Available at: https://socialecology.uci.edu/news/its-not-you-its-your-heritage.

[41] Collins ME, Guo X, Repka MX, Neitzel AJ, Friedman DS. Lessons learned from school-based delivery of vision care in Baltimore, Maryland. Asia Pac J Ophthalmol. 2022;11:6–11. 10.1097/APO.0000000000000488 35066521

[42] Neitzel AJ, Wolf B, Guo X, Shakarchi AF, Madden NA, Repka MX, et al. Effect of a randomized interventional school-based vision program on academic performance of students in grades 3 to 7: a cluster randomized clinical trial. JAMA Ophthalmol. 2021;139:1104–14. 34499111 10.1001/jamaophthalmol.2021.3544 PMC8430909

[43] Frazier M, Garces I, Scarinci I, Marsh-Tootle W. Seeking eye care for children: perceptions among Hispanic immigrant parents. J Immigr Minor Health. 2009;11:215–21. 18551368 10.1007/s10903-008-9160-4

[44] Johnson-Griggs MA, Hicks PM, Lu M-C, Sherman E, Niziol LM, Elam AR, et al. Relationship between unstable housing, food insecurity, and vision status in the Mi-Sight Community Eye Disease Screening Program. Ophthalmology. 2024;131:140–9. 37709171 10.1016/j.ophtha.2023.09.011 PMC11044600

[45] Hu J, Kind AJH, Nerenz D. Area deprivation index (ADI) predicts readmission risk at an urban teaching hospital. Am J Med Qual. 2018;33:493–501. 29357679 10.1177/1062860617753063 PMC6027592

[46] Anon. Accountable Care Organization (ACO) realizing equity, access, and community health (reach) model | CMS. Available at: https://www.cms.gov/newsroom/fact-sheets/accountable-care-organization-aco-realizing-equity-access-and-community-health-reach-model. 10.1001/jamahealthforum.2025.0724 PMC12032566 40279112

