# Supplementary material for: Area Deprivation and Social Vulnerability Are Associated with Pediatric Vision Screening Outcomes in the University of California, Irvine EyeMobile Program
Source: Eye (Lond). 2026 Apr 1;40(9):1325–34. doi: 10.1038/s41433-026-04394-4 (PMC13269760; doi:10.1038/s41433-026-04394-4)
Supplement: Supplementary file 1 — Supplementary Material [file 41433_2026_4394_MOESM1_ESM.docx]

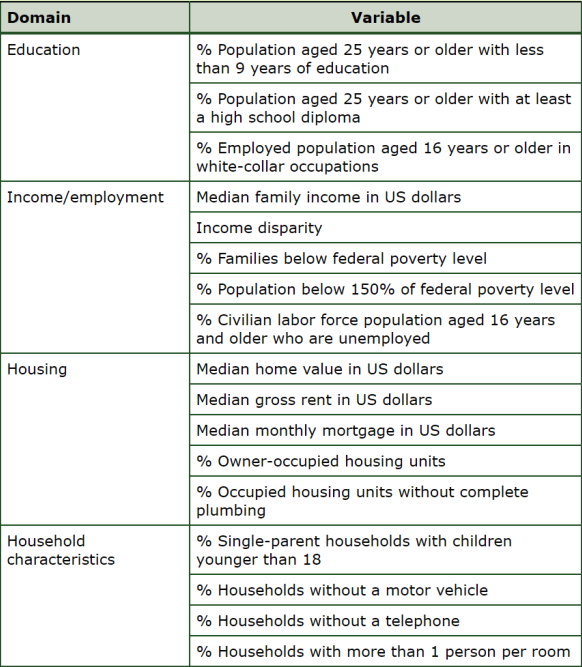


**Supplemental Figure 1.** Components of the Area Deprivation Index (ADI). The figure lists the 17-census block–group indicators (ACS Five-Year Estimates) spanning income, education, employment, and housing quality used to compute the ADI. “Neighborhood” is defined as a Census Block Group; higher national percentiles reflect greater deprivation. In this study, ADI percentiles were grouped into national quartiles (Q1–Q4). The ADI derives from a measure created by HRSA and was refined and validated to the block-group level by Amy Kind, MD, PhD, and colleagues at the University of Wisconsin–Madison. Image reproduced from the Neighborhood Atlas, Center for Health Disparities Research, University of Wisconsin School of Medicine and Public Health (<https://www.neighborhoodatlas.medicine.wisc.edu/>).


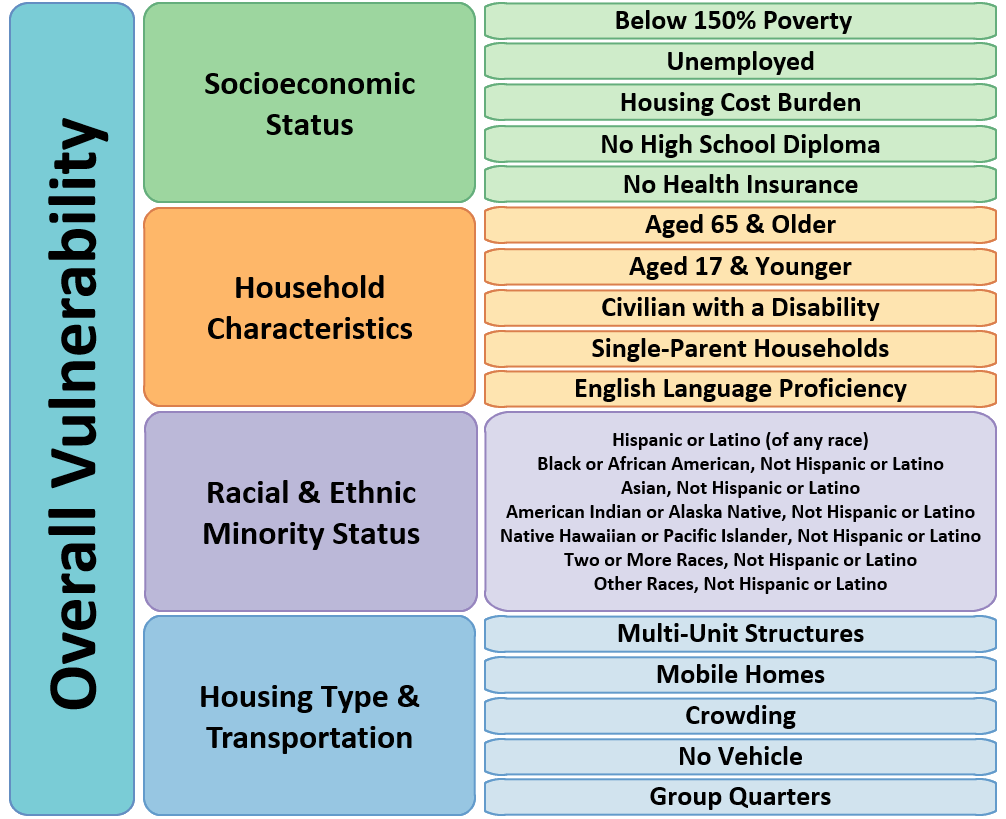


**Supplemental Figure 2.** CDC/ATSDR Social Vulnerability Index (SVI) components. Sixteen 5-year ACS variables, grouped into four themes—Socioeconomic Status; Household Composition & Disability; Minority Status & Language; Housing Type & Transportation are combined into a census-tract–level overall vulnerability score (0–1). The SVI guides preparedness, response, recovery planning, and community-based health promotion initiatives for socially vulnerable communities. Image reproduced from CDC/ATSDR SVI documentation (<https://www.atsdr.cdc.gov/place-health/php/svi/index.html>).

| **Outcome** | **Total** | **ADI Q1**  **(n = 387)** | **ADI Q2**  **(n = 704)** | **ADI Q3**  **(n = 1,065)** | **ADI Q4**  **(n = 1,729)** | **SVI Q1**  **(n = 371)** | **SVI Q2**  **(n = 697)** | **SVI Q3**  **(n = 1076)** | **SVI Q4**  **(n = 1,741)** |
| --- | --- | --- | --- | --- | --- | --- | --- | --- | --- |
| **Normal Dilated Exam** | 1,764 (45.4%) | 220 (56.8%) | 370 (52.6%) | 510 (47.9%) | 664 (38.4%) | 220 (59.3%) | 375 (53.8%) | 500 (46.5%) | 669 (38.4%) |
| **BVAT/Oph Referral** | 198 (5.1%) | 14 (3.6%) | 30 (4.3%) | 54 (5.1%) | 100 (5.8%) | 11 (3.0%) | 24 (3.4%) | 61 (5.7%) | 102 (5.9%) |
| **Non-Dilated Exam** | 751 (19.3%) | 46 (11.9%) | 111 (15.8%) | 181 (17.0%) | 413 (23.9%) | 42 (11.3%) | 107 (15.4%) | 196 (18.2%) | 406 (23.3%) |
| **Fit-for-Frames** | 637 (16.4%) | 60 (15.5%) | 110 (15.6%) | 175 (16.4%) | 292 (16.9%) | 53 (14.3%) | 113 (16.2%) | 175 (16.3%) | 296 (17.0%) |
| **Under Care**^a,b^ | 22 (0.6%) | 2 (0.5%) | 3 (0.4%) | 5 (0.5%) | 12 (0.7%) | 2 (0.5%) | 2 (0.3%) | 6 (0.5%) | 12 (0.7%) |
| **No**  **Show/Consent**^b^ | 513 (13.2%) | 45 (11.6%) | 80 (11.4%) | 140 (13.1%) | 248 (14.3%) | 43 (11.6%) | 76 (10.9%) | 138 (12.8%) | 256 (14.7%) |

a: care of outside medical provider

b: excluded from analysis

**Supplemental Table 1. Clinical examination outcomes among children who underwent comprehensive eye examination, stratified by Area Deprivation Index and Social Vulnerability Index**

Table includes all children referred during initial visit and those who independently scheduled for follow-up visit (n = 3,885). Children listed as “No Show/Consent” or “Under Care” did not complete an EyeMobile examination and were excluded from downstream clinical and regression analyses, yielding 3,350 children with completed examinations. Values are reported as n (%). Quartiles: Q1 = least deprived/vulnerable; Q4 = most deprived/vulnerable. Overall distributions of clinical examination outcomes differed significantly across ADI and SVI quartiles (omnibus χ² p < 0.001 for both indices).

| **Characteristic** | **Screened Cohort (n = 15,842)** | **Examined Cohort (n = 3,350)** | **P-value** |
| --- | --- | --- | --- |
| Age, mean (SD), years | 6.1 (1.9) | 6.2 (1.8) | 0.563 |
| Male sex, n (%) | 8,030 (50.7) | 1,712 (51.1) | 0.675 |
| Female sex, n (%) | 7,812 (49.3) | 1,638 (48.9) | — |
| Race/Ethnicity, n (%) |  |  | 0.932 |
| -Hispanic | 8,191 (51.7) | 1,755 (52.4) |  |
| -Caucasian | 3,723 (23.5) | 765 (22.8) |  |
| -Asian | 2,646 (16.7) | 555 (16.6) |  |
| -Middle Eastern | 887 (5.6) | 190 (5.7) |  |
| -African American | 395 (2.5) | 85 (2.5) |  |
| Passed initial screening, n (%) | 11,199 (70.7) | — | — |
| Referred after screening, n (%) | 3,609 (22.8) | 3,350 (100.0) | — |
| Area Deprivation Index (ADI) |  |  | 0.558 |
| -Quartile 1 (Q1) | 2,155 (13.6) | 472 (14.1) |  |
| -Quartile 2 (Q2) | 2,456 (15.5) | 499 (14.9) |  |
| -Quartile 1 (Q3) | 3,707 (23.4) | 761 (22.7) |  |
| -Quartile 1 (Q4) | 7,524 (47.5) | 1,618 (48.3) |  |
| Social Vulnerability Index (SVI) |  |  | 0.370 |
| -Quartile 1 (Q1) | 2,313 (14.6) | 462 (13.8) |  |
| -Quartile 2 (Q2) | 3,073 (19.4) | 673 (20.1) |  |
| -Quartile 1 (Q3) | 5,133 (32.4) | 1,059 (31.6) |  |
| -Quartile 1 (Q4) | 5,323 (33.6) | 1,156 (34.5) |  |

*Note: P-values reflect comparisons between screened and examined cohorts using t-tests for continuous variables and χ² tests for categorical variables.*

**Supplemental Table 2.** Comparison of children who did and did not undergo comprehensive eye examination

| **Visual Acuity Category (logMAR)** | **Subjects (% total)** | | | | | | | | | | | |
| --- | --- | --- | --- | --- | --- | --- | --- | --- | --- | --- | --- | --- |
| **Uncorrected Distance Visual Acuity (logMAR) by Eye** | **ADI Q1**  **(n=654)** | **ADI Q2**  **(n=820)** | **ADI Q3**  **(n=1101)** | | **ADI Q4**  **(n=2211)** | **Total Eyes**  **(n=4786)** | **SVI Q1**  **(n=693)** | | **SVI Q2**  **(n=1125)** | | **SVI Q3**  **(n=1589)** | **SVI Q4**  **(n=1379)** |
| <0.2 (20/30 or better) | 343 (52.4%) | 452 (55.1%) | 618 (56.1%) | | 1234 (55.8%) | 2647 (55.3%) | 370 (53.4%) | | 617 (54.8%) | | 901 (56.7%) | 759 (55.1%) |
| 0.3–0.4 (20/40 to 20/50) | 190 (29.0%) | 205 (25.0%) | 265 (24.1%) | | 515 (23.3%) | 1175 (24.6%) | 188 (27.1%) | | 293 (26.0%) | | 390 (24.5%) | 304 (22.0%) |
| 0.5–0.6 (20/63 to 20/80) | 89 (13.6%) | 95 (11.6%) | 122 (11.1%) | | 247 (11.2%) | 553 (11.6%) | 84 (12.1%) | | 134 (11.9%) | | 180 (11.3%) | 155 (11.2%) |
| >0.7 (20/100 or worse) | 32 (4.9%) | 68 (8.3%) | 96 (8.7%) | | 215 (9.7%) | 411 (8.6%) | 51 (7.4%) | | 81 (7.2%) | | 118 (7.4%) | 161 (11.7%) |
| **Uncorrected Near Visual Acuity (logMAR) by Eye** | **ADI Q1**  **(n = 651)** | **ADI Q2**  **(n = 766)** | **ADI Q3**  **(n = 1067)** | **ADI Q4**  **(n = 2389)** | | **Total Eyes**  **(n = 4873)** | **SVI Q1**  **(n = 628)** | **SVI Q2**  **(n = 1024)** | | **SVI Q3**  **(n = 1586)** | | **SVI Q4**  **(n = 1635)** |
| <0.2 (20/30 or better) | 375 (57.6%) | 435 (56.8%) | 560 (52.5%) | | 1176 (49.2%) | 2546 (52.2%) | 360 (57.3%) | | 598 (58.4%) | | 820 (51.7%) | 768 (47.0%) |
| 0.3–0.4 (20/40 to 20/50) | 210 (32.3%) | 237 (30.9%) | 300 (28.1%) | | 631 (26.3%) | 1378 (28.3%) | 199 (31.7%) | | 321 (31.3%) | | 443 (27.9%) | 415 (25.4%) |
| 0.5–0.6 (20/63 to 20/80) | 35 (5.4%) | 43 (5.6%) | 120 (11.2%) | | 218 (9.1%) | 416 (8.5%) | 28 (4.5%) | | 53 (5.2%) | | 152 (9.6%) | 183 (11.2%) |
| >0.7 (20/100 or worse) | 31 (4.8%) | 51 (6.7%) | 87 (8.2%) | | 364 (15.2%) | 533 (10.9%) | 41 (6.5%) | | 52 (5.1%) | | 171 (10.8%) | 269 (16.5%) |
| **Best-Corrected Distance Visual Acuity (with Spectacles) logMAR by Eye** | **ADI Q1**  **(n = 153)** | **ADI Q2**  **(n = 162)** | **ADI Q3**  **(n = 853)** | **ADI Q4**  **(n = 1263)** | | **Total Eyes**  **(n = 2431)** | **SVI Q1**  **(n = 317)** | **SVI Q2**  **(n = 655)** | | **SVI Q3**  **(n = 897)** | | **SVI Q4**  **(n = 562)** |
| <0.2 (20/30 or better) | 128 (83.7%) | 142 (87.7%) | 738 (86.5%) | | 1035 (81.9%) | 2043 (84.0%) | 268 (84.5%) | | 556 (84.9%) | | 769 (85.7%) | 450 (80.1%) |
| 0.3–0.4 (20/40 to 20/50) | 20 (13.1%) | 11 (6.8%) | 103 (12.1%) | | 164 (13.0%) | 298 (12.3%) | 40 (12.6%) | | 78 (11.9%) | | 99 (11.0%) | 81 (14.4%) |
| 0.5–0.6 (20/63 to 20/80) | 2 (1.3%) | 4 (2.5%) | 10 (1.2%) | | 43 (3.4%) | 59 (2.4%) | 6 (1.9%) | | 13 (2.0%) | | 18 (2.0%) | 22 (3.9%) |
| >0.7 (20/100 or worse) | 3 (2.0%) | 5 (3.1%) | 2 (0.2%) | | 21 (1.7%) | 31 (1.3%) | 3 (0.9%) | | 8 (1.2%) | | 11 (1.2%) | 9 (1.6%) |

**Supplemental Table 3. Uncorrected and best-corrected distance and near visual acuity by eye, stratified by Area Deprivation Index and Social Vulnerability Index quartiles**

Visual acuity outcomes from comprehensive ophthalmologic examinations are categorized by logMAR equivalents and stratified by quartiles of the ADI and SVI where Quartile 1 (Q1) represents the least deprived or vulnerable communities and Quartile 4 (Q4) the most deprived or vulnerable. Each visual acuity domain, uncorrected distance, uncorrected near, and best-corrected distance visual acuity, is presented within a single table. Percentages are calculated row-wise within each acuity domain and quartile, reflecting the distribution of acuity levels among eyes examined in that subgroup.

BCVA was the prespecified primary visual outcome. For BCVA, distributions differed significantly across quartiles for both ADI and SVI (overall χ² p < 0.001 for each). Significant monotonic increases in poor BCVA across increasing quartiles were also observed (Cochran–Armitage trend p < 0.001 for ADI and SVI).

The number of eyes contributing to each domain differs due to age-appropriate testing, cooperation, and examination completion in a school-based mobile clinic setting (uncorrected distance VA: n = 4,786 eyes; uncorrected near VA: n = 4,873 eyes; BCVA: n = 2,431 eyes). All children contributing data to this table underwent comprehensive ophthalmologic examination following school-based vision screening referral.

| **Refractive Error Classification** | **Subjects (% total)** | | | | | | | | |
| --- | --- | --- | --- | --- | --- | --- | --- | --- | --- |
| **Myopia (\|SE\|, D)** | **ADI Q1 (n=405)** | **ADI Q2**  **(n=380)** | **ADI Q3 (n=320)** | **ADI Q4 (n=285)** | **Total Eyes**  **(n=1390)** | **SVI Q1 (n=445)** | **SVI Q2 (n=390)** | **SVI Q3 (n=301)** | **SVI Q4 (n=254)** |
| 0.50 to 1.75 | 302 (74.6%) | 283 (74.5%) | 240 (75.0%) | 213 (74.7%) | 1,038 | 332 (74.6%) | 291 (74.6%) | 224 (74.7%) | 191 (74.5%) |
| 2.00 to 3.75 | 79 (19.5%) | 74 (19.5%) | 62 (19.4%) | 55 (19.3%) | 270 | 86 (19.3%) | 76 (19.5%) | 58 (19.3%) | 50 (19.6%) |
| 4.00 to 5.75 | 21 (5.2%) | 19 (5.0%) | 15 (4.7%) | 13 (4.6%) | 68 | 24 (5.4%) | 19 (4.9%) | 15 (5.0%) | 10 (4.7%) |
| ≥ 6.00 | 3 (0.7%) | 4 (1.1%) | 3 (0.9%) | 4 (1.4%) | 14 | 3 (0.7%) | 4 (1.0%) | 4 (1.0%) | 3 (1.2%) |
| **with Astigmatism** | 354 (87.4%) | 330 (86.8%) | 296 (92.5%) | 253 (88.8%) | 1,233 | 377 (84.7%) | 332 (85.1%) | 288 (95.7%) | 236 (92.9%) |
| **Hyperopia (\|SE\|, D)** | **ADI Q1 (n=531)** | **ADI Q2**  **(n=496)** | **ADI Q3 (n=480)** | **ADI Q4 (n=501)** | **Total Eyes**  **(n=2008)** | **SVI Q1 (n=443)** | **SVI Q2 (n=481)** | **SVI Q3 (n=542)** | **SVI Q4 (n=542)** |
| 0.50 to 1.75 | 378 (71.2%) | 350 (70.6%) | 342 (71.2%) | 356 (71.0%) | 1,426 | 314 (71.0%) | 342 (71.1%) | 385 (71.0%) | 385 (71.0%) |
| 2.00 to 3.75 | 119 (22.4%) | 114 (23.0%) | 107 (22.3%) | 112 (22.4%) | 452 | 100 (22.6%) | 108 (22.5%) | 122 (22.5%) | 122 (22.5%) |
| 4.00 to 5.75 | 25 (4.7%) | 24 (4.8%) | 23 (4.8%) | 24 (4.8%) | 96 | 21 (4.7%) | 23 (4.8%) | 26 (4.8%) | 26 (4.8%) |
| ≥ 6.00 | 9 (1.7%) | 8 (1.6%) | 8 (1.7%) | 9 (1.8%) | 34 | 8 (1.8%) | 8 (1.7%) | 9 (1.7%) | 9 (1.7%) |
| **with Astigmatism** | 406 (76.4%) | 390 (77.7%) | 375 (78.1%) | 390 (77.8%) | 1,561 | 343 (77.4%) | 375 (77.9%) | 421 (77.7%) | 422 (77.9%) |
| **Astigmatism (\|Cyl\|, D)** | **ADI Q1 (n=594)** | **ADI Q2**  **(n=792)** | **ADI Q3 (n=1,149)** | **ADI Q4 (n=1,426)** | **Total Eyes**  **(n=3961)** | **SVI Q1 (n=753)** | **SVI Q2 (n=911)** | **SVI Q3 (n=1,069)** | **SVI Q4 (n=1,228)** |
| 0.50 to 1.75 | 353 (59.4%) | 471 (59.5%) | 683 (59.4%) | 848 (59.5%) | 2,355 | 448 (59.5%) | 542 (59.5%) | 636 (59.5%) | 729 (59.4%) |
| 2.00 to 3.75 | 197 (33.2%) | 263 (33.2%) | 381 (33.2%) | 474 (33.2%) | 1,315 | 250 (33.2%) | 302 (33.2%) | 355 (33.2%) | 408 (33.2%) |
| 4.00 to 5.75 | 41 (6.9%) | 54 (6.8%) | 79 (6.9%) | 97 (6.8%) | 271 | 51 (6.8%) | 62 (6.8%) | 73 (6.8%) | 85 (6.9%) |
| ≥ 6.00 | 3 (0.5%) | 4 (0.5%) | 6 (0.5%) | 7 (0.5%) | 20 | 4 (0.5%) | 5 (0.5%) | 5 (0.5%) | 6 (0.5%) |
| ***Anisometropia (\|ASE\| ≥ 1.00 D)** | **ADI Q1 (31/641)** | **ADI Q2**  **(47/672)** | **ADI Q3 (63/670)** | **ADI Q4 (105/709)** | **Total Eyes**  **(n=246/2692)** | **SVI Q1 (33/618)** | **SVI Q2 (53/661)** | **SVI Q3 (69/721)** | **SVI Q4 (91/692)** |
| ΔSph ≥ 1.00 D | 25 (80.6%) | 39 (83.0%) | 54 (85.7%) | 85 (81.0%) | 203 | 27 (81.8%) | 41 (77.4%) | 56 (81.2%) | 79 (86.8%) |
| ΔCYL ≥ 1.00 D | 12 (38.7%) | 18 (38.3%) | 23 (36.5%) | 38 (36.2%) | 91 | 13 (39.4%) | 19 (35.8%) | 24 (34.8%) | 35 (38.5%) |
| with Myopia | 11 (10.3%) | 22 (20.6%) | 30 (28.0%) | 44 (41.1%) | 107 | 13 (12.1%) | 22 (20.6%) | 30 (28.0%) | 42 (39.3%) |
| with Hyperopia | 17 (10.1%) | 32 (19.0%) | 50 (29.8%) | 69 (41.1%) | 168 | 21 (12.4%) | 34 (20.1%) | 48 (28.4%) | 66 (39.1%) |
| with Astigmatism | 22 (10.0%) | 48 (21.9%) | 59 (27.0%) | 90 (41.1%) | 219 | 27 (12.3%) | 45 (20.5%) | 62 (28.3%) | 85 (38.8%) |
| with Myopia and Astigmatism | 11 (10.3%) | 19 (17.8%) | 33 (30.8%) | 44 (41.1%) | 107 | 13 (12.1%) | 22 (20.6%) | 30 (28.0%) | 42 (39.3%) |
| with Hyperopia and Astigmatism | 17 (10.1%) | 34 (20.2%) | 48 (28.6%) | 69 (41.1%) | 168 | 21 (12.4%) | 34 (20.1%) | 48 (28.4%) | 66 (39.1%) |
| **Emmetropia** | **ADI Q1**  **(n=1050)** | **ADI Q2**  **(n=1150)** | **ADI Q3**  **(n=1300)** | **ADI Q4**  **(n=1884)** | **Total Eyes**  **(n=5384)** | **SVI Q1**  **(n=1100)** | **SVI Q2**  **(n=1200)** | **SVI Q3**  **(n=1450)** | **SVI Q4**  **(n=1634)** |
|  | 420 (40.0%) | 455 (39.6%) | 500 (38.5%) | 611 (32.4%) | 1,986 (36.9%) | 440 (40.0%) | 468 (39.0%) | 527 (36.3%) | 551 (33.7%) |

**Anisometropia analyses were restricted to eyes with complete paired refraction data, resulting in denominators that differ from refractive error–specific totals shown elsewhere in the table. For example, in ADI Q1, 31 anisometropic eyes were identified among 641 eyes evaluable for anisometropia. Subcategories (e.g., spherical difference, cylindrical difference, with myopia, with hyperopia, with astigmatism) are not mutually exclusive; individual eyes may be counted in multiple categories. Overall, 246 of 2,692 eyes (9.1%) met criteria for anisometropia.*

**Supplemental Table 4.** Refractive error classification stratified by Area Deprivation Index and Social Vulnerability Index quartiles

Refraction outcomes were categorized by spherical equivalent (SE), cylindrical power (Cyl), and anisometropia (ASE), and stratified across quartiles of the Area Deprivation Index (ADI) and Social Vulnerability Index (SVI), where Quartile 1 (Q1) represents the least deprived or vulnerable communities and Quartile 4 (Q4) the most deprived or vulnerable. Values are reported as number of eyes and row-wise percentages within each refractive error category and quartile.

Overall distributions of refractive error classifications differed significantly across both ADI and SVI quartiles (overall χ² p < 0.001 for myopia and astigmatism for both indices). Myopia prevalence decreased monotonically with increasing neighborhood disadvantage, whereas astigmatism prevalence increased across quartiles (Cochran–Armitage trend p < 0.001 for both ADI and SVI). Hyperopia and anisometropia did not demonstrate significant differences across quartiles (overall χ² p > 0.05 for both ADI and SVI).

Analyses were conducted at the eye level. Broad refractive error categories (myopia, hyperopia, emmetropia) were derived from eyes with sufficient and complete refraction data, including both cycloplegic and non-cycloplegic measurements (n = 5,384 eyes). In contrast, analyses requiring precise refractive measurements in downstream outcomes (including BCVA and amblyopia suspect classification) were restricted to eyes with cycloplegic refraction only (n = 5,198 eyes). Anisometropia analyses were further restricted to eyes with complete paired cycloplegic refraction data (n = 2,692 eyes).

| **Model** | **Predictor / Group** | **aOR** | **95% CI** | **p-value** |
| --- | --- | --- | --- | --- |
| **Model 1: ADI (adjusted for race/ethnicity and gender)** |  |  |  |  |
|  | ADI Q4 vs Q1 | 1.51 | 1.13–2.01 | 0.006 |
|  | Hispanic vs White | 1.73 | 1.22–2.41 | 0.002 |
|  | Asian vs White | 1.35 | 0.91–1.71 | 0.240 |
|  | Female vs Male | 1.25 | 1.01–1.55 | 0.041 |
|  | Race × ADI interaction | — | — | <0.01 |
| **Stratified by Race (ADI Q4 vs Q1)** |  |  |  |  |
|  | Hispanic | 1.87 | 1.38–2.79 | 0.002 |
|  | Asian | 1.62 | 1.05–2.21 | 0.033 |
|  | White | 1.06 | 0.72–1.55 | 0.640 |
| **Model 2: SVI (adjusted for race/ethnicity and gender)** |  |  |  |  |
|  | SVI Q4 vs Q1 | 1.61 | 1.18–2.20 | 0.002 |
|  | Hispanic vs White | 1.73 | 1.22–2.41 | 0.002 |
|  | Asian vs White | 1.35 | 0.91–1.71 | 0.240 |
|  | Female vs Male | 1.25 | 1.01–1.55 | 0.041 |
|  | Race × SVI interaction | — | — | 0.020 |
| **Stratified by Race (SVI Q4 vs Q1)** |  |  |  |  |
|  | Hispanic | 1.91 | 1.31–2.78 | 0.001 |
|  | Asian | 1.44 | 1.09–2.05 | 0.046 |
|  | White | 1.14 | 0.81–1.60 | 0.450 |

*Note: Separate multivariable regression models were fit for ADI and SVI to avoid multicollinearity due to overlapping neighborhood constructs. Interaction terms (Race×ADI or Race×SVI) were tested within index-specific models.*

**Supplemental Table 5.** Multivariable and stratified logistic regression predicting poor best-corrected visual acuity (BCVA > 0.2 logMAR)

Combined results from multivariable and stratified logistic regression models. Separate multivariable models were constructed for ADI and for SVI, with interaction terms tested (Race×ADI or Race×SVI, as appropriate). Stratified models estimate the adjusted odds of poor BCVA for Quartile 4 versus Quartile 1 within racial/ethnic groups, adjusting for age, sex, and refractive error type and severity. aOR = adjusted odds ratio; CI = confidence interval.

| **Index** | **Quartile** | **Amblyopia Suspect / Total Children Examined (n)** | **% Amblyopia Suspect** |
| --- | --- | --- | --- |
| **ADI** | Q1 | 28/387 | 7.2% |
|  | Q2 | 56/704 | 8.0% |
|  | Q3 | 102/1065 | 9.6% |
|  | Q4 | 148/1194 | 12.4% |
| **SVI** | Q1 | 27/360 | 7.5% |
|  | Q2 | 51/680 | 7.5% |
|  | Q3 | 98/1040 | 9.4% |
|  | Q4 | 150/1270 | 11.8% |
| **Total** |  | 334 / 3,350 | 10.0% |

**Supplemental Table 6.** Prevalence of amblyopia suspect by Area Deprivation Index and Social Vulnerability Index quartiles (n=3,350 children examined; 334 children diagnosed with amblyopia suspect)
